# Validation of Ultrasound for Quantification of Knee Meniscal Tissue: A Cadaveric Study

**DOI:** 10.3390/diagnostics15030389

**Published:** 2025-02-06

**Authors:** Jacobo Rodríguez-Sanz, Miguel Malo-Urriés, Sergio Borrella-Andrés, Isabel Albarova-Corral, Carlos López-de-Celis

**Affiliations:** 1 Faculty of Medicine and Health Science, Universitat Internacional de Catalunya, 08195 Barcelona, Spain; jrodriguezs@uic.es (J.R.-S.); carlesldc@uic.es (C.L.-d.-C.); 2 ACTIUM Functional Anatomy Group, 08195 Barcelona, Spain; 3 PhysiUZerapy Health Sciences Research Group, Health Sciences Faculty, Department of Physiatry and Nursing, University of Zaragoza, 50009 Zaragoza, Spain; sergiocai04@gmail.com (S.B.-A.); ialbarova@unizar.es (I.A.-C.); 4 Fundació Institut Universitari per a la Recerca a l’Atenció Primària de Salut Jordi Gol i Gurina, 08035 Barcelona, Spain

**Keywords:** ultrasound, meniscus, cadaver, validation, knee

## Abstract

****Background**:** While MRI is the gold standard for meniscal assessment, its cost and accessibility limitations have led to growing interest in ultrasound, though its validity for quantifying meniscal tissue remains unclear. To validate the use of ultrasound in quantifying meniscal tissue across the anterior, middle, and posterior regions of both menisci (medial and lateral) in longitudinal and transverse planes by comparison with cadaveric dissection. **Methods**: A cross-sectional study was conducted on ten cryopreserved anatomical donors, obtaining a total of 120 ultrasound scans from the different meniscal regions. Following ultrasound imaging, cadaveric dissection was performed to facilitate photometric measurements, thereby enabling validation of the ultrasound findings. The intra-examiner reliability of the ultrasound measurements was also assessed. **Results**: The intra-examiner reliability of ultrasound measurements ranged from moderate to excellent. A strong and statistically significant positive correlation was observed between ultrasound and photometric measurements across all meniscal regions (r > 0.821; *p* < 0.05). In the medial meniscus, ultrasound visualized 99.1% of the anterior region (8.71 mm with ultrasound; 8.64 mm with photometry), 96.3% of the middle region (9.09 mm with ultrasound; 9.39 mm with photometry), and 98.5% of the posterior region (10.54 mm with ultrasound; 10.61 mm with photometry). In the lateral meniscus, ultrasound visualized 107.1% of the anterior region, 105.1% of the middle region, and 97.8% of the posterior region. The observed excess in tissue visualization in some regions likely reflects the inclusion of adjacent connective tissue, indistinguishable from meniscal tissue on ultrasound. **Conclusions**: Ultrasound is a valid and reliable modality for visualizing most meniscal tissue across regions, with a measurement discrepancy under 0.7 mm compared to anatomical dissection. However, caution is advised as adjacent connective tissue may sometimes be misidentified as meniscal tissue during evaluations.

## 1. Introduction

The menisci are two crescent-shaped, fibrocartilaginous disks positioned between the femoral condyles and the proximal tibia on the medial and lateral sides of the knee joint [1]. These intra-articular structures are pivotal in knee biomechanics, aiding in load distribution, shock absorption, lubrication, and joint stability [2]. Anatomically, the meniscus is a wedge-shaped, semilunar structure with a thicker outer edge and a thinner, tapering central portion. Its superior surface is concave to fit the femoral condyle, while the flat base attaches to the tibia through a central root ligament, which is crucial for its function and load distribution across the tibial plateau [3]. The vascular supply to the meniscus is restricted to its peripheral third, creating three distinct zones that influence its healing potential: the anterior zone (vascular), the intermediate zone, and the posterior zone (avascular) [4]. Meniscal injuries are of significant concern due to their high incidence and impact on knee function and overall joint health. The socioeconomic impact of meniscal injuries is substantial, encompassing direct medical costs such as surgical procedures and rehabilitation, as well as indirect costs like loss of productivity and long-term disability [5]. Furthermore, meniscal injuries are a leading cause of knee osteoarthritis in the long term, contributing to increased healthcare utilization and diminished quality of life for affected individuals [6]. Understanding the epidemiology and socioeconomic burden of meniscal injuries underscores the need for effective diagnostic and therapeutic strategies to mitigate their impact on individuals and healthcare systems.

Traditionally, meniscal injury evaluation involves clinical examination followed by imaging modalities like magnetic resonance imaging (MRI) and arthroscopy. MRI is considered the gold standard for diagnosing meniscal pathology due to its high sensitivity and specificity [7]. Recent meta-analytic data reinforce this, demonstrating that MRI has a global sensitivity and specificity of 92.0% and 90.0% for medial meniscal tears and 80.0% and 95.0% for lateral meniscal tears, respectively [8]. However, MRI has its limitations, including high costs, limited availability, contraindications for certain patients, and the inability to provide real-time dynamic assessment. Furthermore, MRI’s diagnostic accuracy can be compromised by artifacts and the need for static imaging, which does not capture joint dynamics.

Ultrasound (US) has emerged as a valuable diagnostic tool for musculoskeletal conditions, offering several advantages over MRI, such as lower costs, portability, real-time dynamic assessment, and fewer contraindications [9]. Recent advancements in US technology have enhanced its application in evaluating meniscal tissue [10]. Invasive techniques, such as US-guided interventions, have potential in regard to precise treatment delivery to various meniscal regions. These techniques can potentially enhance the accuracy and efficacy of meniscal repair and pain management strategies [11,12]. However, despite these promising developments, the scientific evidence supporting the use of US for comprehensive meniscal evaluation and treatment guidance remains limited, requiring further validation through rigorous research.

The present study aims to validate the use of ultrasound in the measurement of the tissue of both menisci in the longitudinal and transverse planes of the anterior, as well as the middle and posterior regions, followed by cadaveric dissection to validate the ultrasound findings through comparison with the anatomical reality. This research seeks to contribute to the growing body of evidence supporting the use of ultrasound for meniscal assessment and intervention, ultimately enhancing clinical practice and patient outcomes.

## 2. Material and Methods

### 2.1. Cadaveric Sample

A cross-sectional study was designed to determine the validity of ultrasound in the visualization of the knee meniscus and to quantify the percentage of the meniscus that can be visualized. The gold standard for comparison was dissection performed subsequently.

A total of 120 meniscal ultrasound scans were performed on ten cryopreserved anatomical donors at the anatomy laboratory of the Universitat Internacional de Catalunya (Spain). The 120 ultrasound scans were obtained by performing 10 longitudinal and 10 transverse ultrasound scans of the following regions of the menisci:

- Medial meniscus (anterior region: n = 20; middle region: n = 20; posterior region: n = 20).
- Lateral meniscus (anterior region: n = 20; middle region: n = 20; posterior region: n = 20).

To determine the suitability of the bodies for inclusion in the present study, a visual examination was conducted to confirm the absence of scars in the knee region as well as any evidence of knee injury or prior surgical procedures. The bodies were stored at −20 °C and acclimatized to room temperature 48 h before the start of the investigation. This study was approved by the Local Ethics Committee of the Center (CBAS-2024-06).

### 2.2. Sample Size

The sample size was calculated using G*Power V.3.1.9.6, assuming an infinite population. An exact family, correlation, and bivariate test was applied. The analysis was performed “a priori” with a minimum target correlation of 0.7. The α risk was set at 0.05 (two-sided test) and the β risk at 0.05.

The result indicated that 20 ultrasound scans were required for each part of the meniscus. The study aimed to analyze the anterior, middle, and posterior regions of both the medial and lateral menisci, resulting in a total of 120 ultrasound scans needed to complete the study.

### 2.3. Ultrasound-Guided Approach

The procedures were conducted by a therapist with over 10 years of experience in ultrasound techniques. The objective was to visualize as much of the meniscus as possible using ultrasound, followed by verification through cadaveric dissection. Ultrasound imaging was performed using a General Electric LOGIQ e R9 device equipped with a linear transducer (4–20 MHz).

Anterior Region of the Meniscus

To evaluate the anterior region of the meniscus, the patient was placed in a supine position. The optimal knee flexion–extension angle for visualizing the meniscus was identified. Additionally, depending on whether the anterior part of the medial or lateral meniscus was being examined, a medial or lateral rotation of the tibia was applied, respectively.

**Figure 1:**
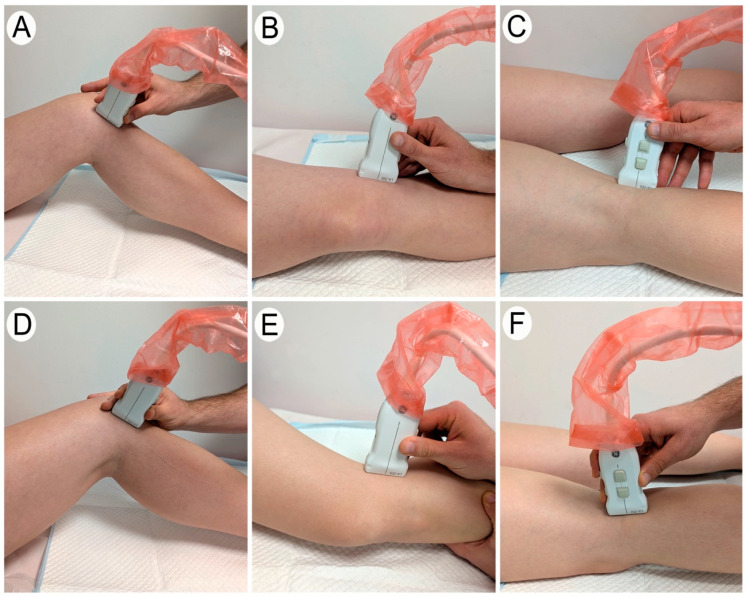
A transverse ultrasound scan of the medial and lateral meniscus. The images show the appropriate transducer placement to obtain a transverse view of each meniscal region. (**A**–**C**) Transverse ultrasound imaging of the medial meniscus: the anterior (**A**), middle (**B**), and posterior (**C**) regions. (**D**–**F**) Transverse ultrasound imaging of the lateral meniscus: the anterior (**D**), middle (**E**), and posterior (**F**) regions.

**Figure 2:**
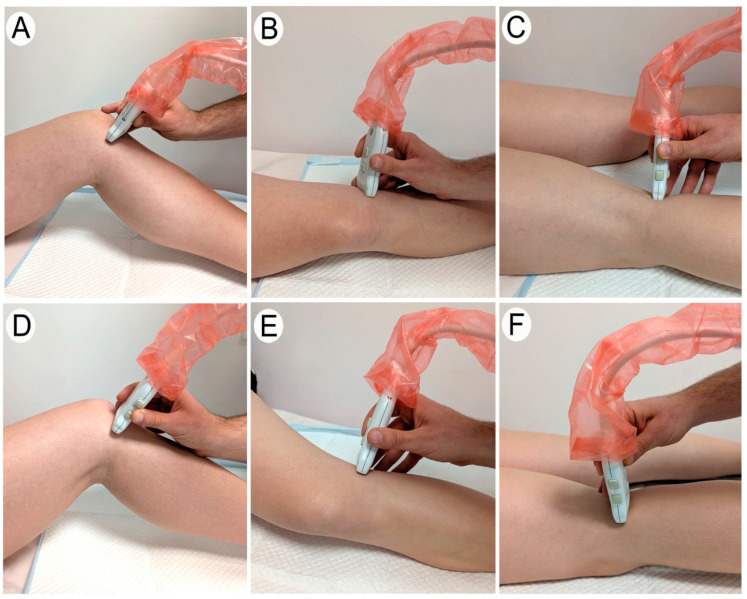
A longitudinal ultrasound scan of the medial and lateral meniscus. The images show the appropriate transducer placement to obtain a longitudinal view of each meniscal region. (**A**–**C**) Longitudinal ultrasound imaging of the medial meniscus: the anterior (**A**), middle (**B**), and posterior (**C**) regions. (**D**–**F**) Longitudinal ultrasound imaging of the lateral meniscus: the anterior (**D**), middle (**E**), and posterior (**F**) regions.

**Figure 3:**
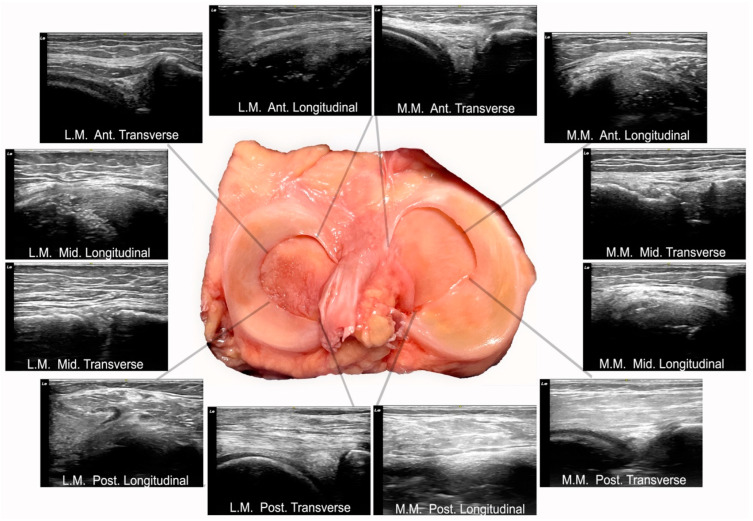
Longitudinal and transverse ultrasound scans of each meniscus region. Abbreviations: M.M, medial meniscus; L.M, lateral meniscus; Ant, anterior; Mid, middle; Post, posterior.

The examination began with a longitudinal scan of the patellar tendon. For the medial meniscus, the transducer was then moved medially. As the transducer was shifted, the image of the patellar tendon gradually disappeared, revealing only fatty tissue and the cortical bone of the tibia and femur. Upon reaching the anterior region of the medial meniscus, a more hyperechoic structure located between the tibia and femur was observed (Figure 1). This structure corresponded to the anterior region of medial meniscus, viewed in a transverse plane. At this point, the evaluator identified the position where the largest portion of the meniscus could be visualized and saved the image for later measurement. Subsequently, the transducer was reoriented to visualize the same region of the meniscus in a longitudinal plane (Figure 2). This image was also saved for subsequent measurement (Figure 3).

The procedure for visualizing the anterior region of the lateral meniscus followed the same steps, with the transducer being shifted laterally to the patellar tendon (Figure 1, Figure 2 and Figure 3).

Middle Region of the Meniscus

For the exploration of the middle region of the meniscus, the patient was positioned in lateral decubitus. A semi-maximal knee extension angle was selected based on the optimal visualization of the meniscus, and the degree of this final position was recorded using goniometry. Additionally, depending on whether the medial or lateral meniscus was being examined, a lateral or medial rotation of the tibia was applied, respectively.

For the evaluation of the middle part of the medial meniscus, the medial aspects of the femur and tibia were manually palpated. The transducer was then positioned to obtain a longitudinal view of the medial collateral ligament, with the middle part of the medial meniscus visualized just beneath it in a transverse section (Figure 1). The tibia and femur were visible on either side of the meniscal tissue. At this point, the evaluator identified the region where the largest amount of meniscal tissue was visible and saved the image for subsequent measurement. The transducer was then adjusted to obtain a longitudinal view of this meniscal region, and this image was also saved for later measurement (Figure 2).

For the lateral meniscus, a similar procedure was followed, with the only difference being that the transducer was positioned to obtain a longitudinal view of the lateral collateral ligament instead of the medial (Figure 1, Figure 2 and Figure 3).

Posterior Region of the Meniscus

To evaluate the posterior region of the meniscus, the patient was placed in a prone position. The knee was maintained near maximum extension, and the evaluator adjusted the position until optimal visualization of the posterior region of the meniscus was achieved with the ultrasound transducer. Once the position was identified, the degree of extension was recorded using goniometry.

The ultrasound examination began by placing the transducer in a longitudinal section of the popliteal fossa. To locate the posterior region of the medial meniscus in a transverse section, the transducer was then moved medially until an image displaying the femur, tibia, posterior region of the medial meniscus in the center, and part of the lateral gastrocnemius above was observed (Figure 1). At this point, the evaluator identified the image where the greatest amount of the meniscus was visible and saved it for subsequent measurement. Finally, the evaluator adjusted the transducer at the same point to visualize the meniscus in a longitudinal section and saved the image for subsequent measurement (Figure 2).

The process to visualize the posterior region of the lateral meniscus was the same as described above but with the transducer shifted laterally in the popliteal fossa (Figure 1, Figure 2 and Figure 3).

In all images, three measurements were taken to obtain the mean meniscal thickness (Figure 3).

### 2.4. Ultrasonographic Measurements

For each meniscus, three sections were obtained from the anterior, middle, and posterior regions, with both transverse and longitudinal images being captured for each section. Three measurements of the meniscus thickness were taken from each image, and the mean of these three measurements was used for statistical analysis. The measurements were conducted to capture the full thickness of the meniscus, starting from the superficial connective tissue layers corresponding to the capsule (excluded from the measurement) and ranging to the visible free edge of the meniscus. In the transverse sections, measurements were taken parallel to the tibial plane to ensure consistency with the subsequent photographic measurements. In the longitudinal sections, the measurements were performed following a radial alignment from the outer edge of the meniscus toward its center, perpendicular to the meniscus’s curvature. This approach was critical to ensuring accuracy and comparability between the ultrasonographic and photometric measurements.

### 2.5. Anatomical Procedure

The anatomical dissection was performed by an experienced therapist specializing in dissection who was blinded to the previous ultrasound measurements. Dissection was conducted on the knees of body donors until both menisci were fully exposed (Figure 1). Upon completion of the dissection, photographs were taken for subsequent measurement. The measurements were conducted using photometric analyses.

### 2.6. Meniscus Measurement Procedure: Photometry

Once the knee was dissected, exposing a complete view of the meniscus, a photograph was taken perpendicular to the tibial plateau. The images were then measured using photometry by a single investigator. The images were exported in JPG format and analyzed with ImageJ software. 1.54k version (https://imagej.nih.gov/ij/docs/guide/, accessed on 15 July 2024).

Each meniscus was divided into three zones (anterior, middle, and posterior), and each zone was further subdivided into three portions. For each portion and zone, three measurements of the meniscus width were taken, resulting in three measurements per zone (anterior, middle, posterior). To assess the reliability of these measurements, they were repeated two days later following the same procedure.

For statistical analysis, the mean of the measurements recorded in both trials was used for each area.

### 2.7. Meniscus Measurement Procedure: Ultrasound

The same procedure as in photometry was followed, dividing the meniscus into three zones (anterior, middle, and posterior) and performing three measurements corresponding to each third of each zone.

**Figure 4:**
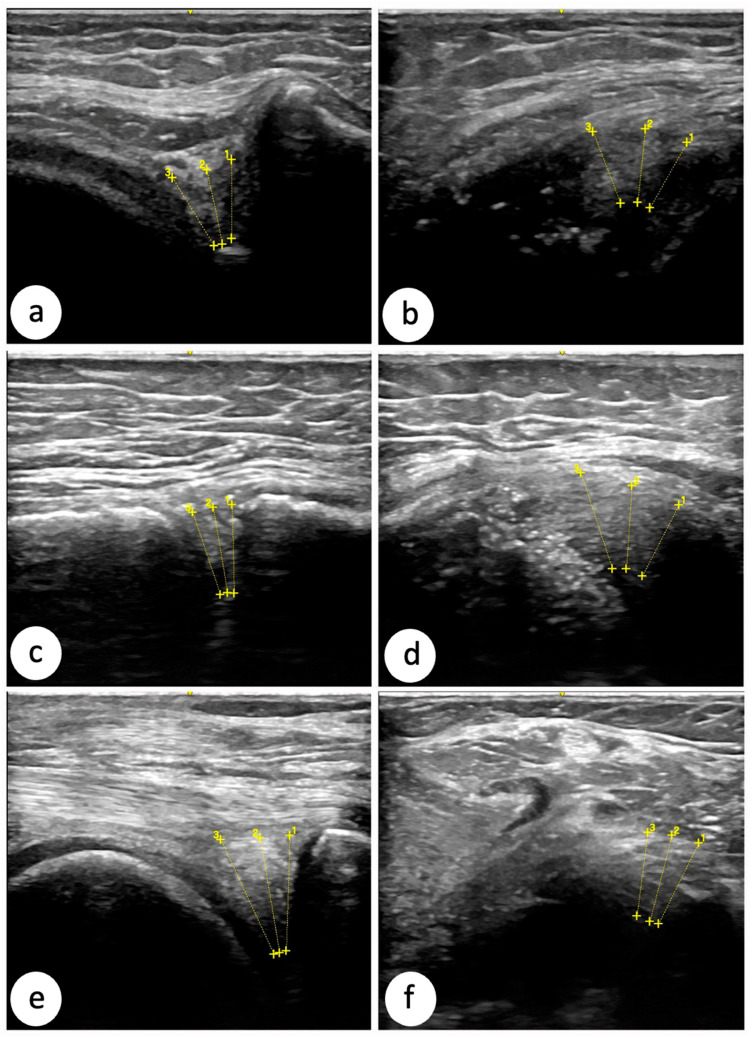
Longitudinal and transverse ultrasound scans of each meniscus region with examples of ultrasound measurements. (**a**) The transverse section of the anterior region of the meniscus; (**b**) longitudinal section of the anterior region of the meniscus; (**c**) transverse section of the middle region of the meniscus; (**d**) longitudinal section of the middle region of the meniscus; (**e**) transverse section of the posterior region of the meniscus; (**f**) longitudinal section of the posterior region of the meniscus.

In the ultrasound measurements, both longitudinal and transverse images were obtained for each zone, and the average of the two measurements was calculated to facilitate a comparative analysis against the photometric data (Figure 4).

All ultrasound and photometric measurements were performed twice, with a one-month interval occurring between the two sets of measurements. This was undertaken to assess the reliability of the researchers in performing both types of measurements.

### 2.8. Statistical Analysis

Statistical analysis was carried out with SPSS v.25 Statistics software (IBM, Armonk, NY, USA) for Windows. The Shapiro–Wilks test was used to test the normality of the data. Descriptive statistics (mean and standard deviations or number and percentage) were calculated to describe the demographic characteristics of the sample. The test–retest reliability of the ultrasounds was calculated using the intraclass correlation coefficient (ICC_2.1_) (two-way random single measures), standard error of measurement (SEM), and the minimal detectable change 95% confidence interval (MDC-95%). To achieve this, the evaluator measured all the ultrasounds and reassessed them after 1 month. For the interpretation of ICC_2.1_, values greater than 0.75 were considered excellent, between 0.4 and 0.75 were moderate, and values less than 0.4 indicated poor reliability. The agreement between the ultrasound and the dissection procedure (gold standard) was examined using Bland and Altman analysis [13] (95% limits of confidence). A Pearson correlation coefficient between the ultrasound and dissection procedure was also performed. A *p* value < 0.05 was considered statistically significant.

## 3. Results

The reliability between the two sets of measurements was very high for both ultrasound and photometry, with values exceeding 0.93 in all regions of the meniscus (Table 1).

Table 2 shows the final data means used for the analysis. The photometry data of the dissections of the anatomical pieces were obtained from the mean of the two measurements taken at different times by the examiner.

The ultrasound data were obtained by calculating the averages of the measurements in the longitudinal section with the transverse section. These measurements were also performed twice at different time points, as previously detailed in the methodology of the study.

The reliability between ultrasound and photometric measurements is shown in Table 3.

We found excellent reliability between the ultrasound and photometry measurements for all meniscal regions except for the anterior and medial zone of the lateral meniscus, where we found moderate reliability.

**Figure 5:**
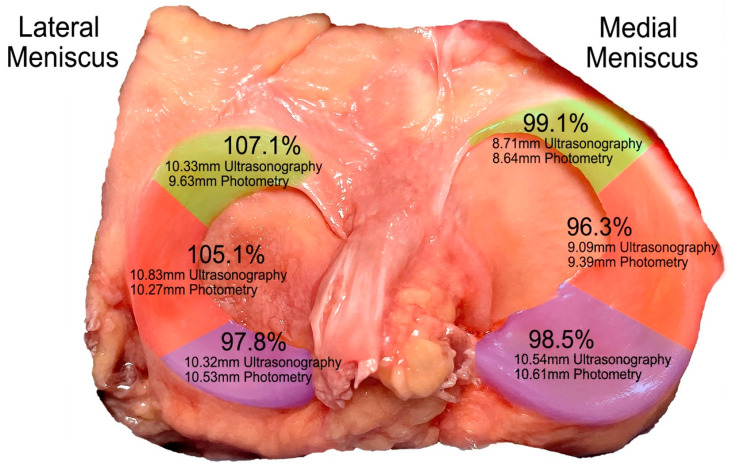
The concordance percentages and millimeters of the photometry and ultrasound techniques used on the different parts of the meniscus. Green: anterior region; Orange: middle region; Purple: posterior region.

The percentage of meniscus visibility was calculated by taking the mean of the longitudinal and transverse measurements of each region. Considering the photometry values as representing 100% of the meniscus, nearly the entire meniscus is visible (Figure 5). Adjusting the ultrasound measurement by subtracting the measurement errors from both ultrasonography and photometry reveals that, in the medial meniscus, visibility ranges from 96.3% to 99.1%, while, in the lateral meniscus, visibility exceeds 97.8% in the posterior region. In the anterior and middle regions of the lateral meniscus, the values exceed 100% (107.1% for the anterior region and 105.1% for the middle region).

The results of the correlation between ultrasound measurements and photometry showed an excellent and statistically significant positive correlation (Pearson) in all regions of the meniscus. For the anterior medial meniscus, the results were r = 0.957; *p* < 0.001; for the middle medial meniscus, the results were r = 0.912; *p* < 0.001); for the posterior medial meniscus, the results were r = 0.854; *p* = 0.002; for the anterior lateral meniscus, the results were r = 0.821; *p* = 0.004; for the middle lateral meniscus, the results were r = 0.880; *p* = 0.001; and for the posterior lateral meniscus, the results were = 0.866; *p* = 0.001).

**Figure 6:**
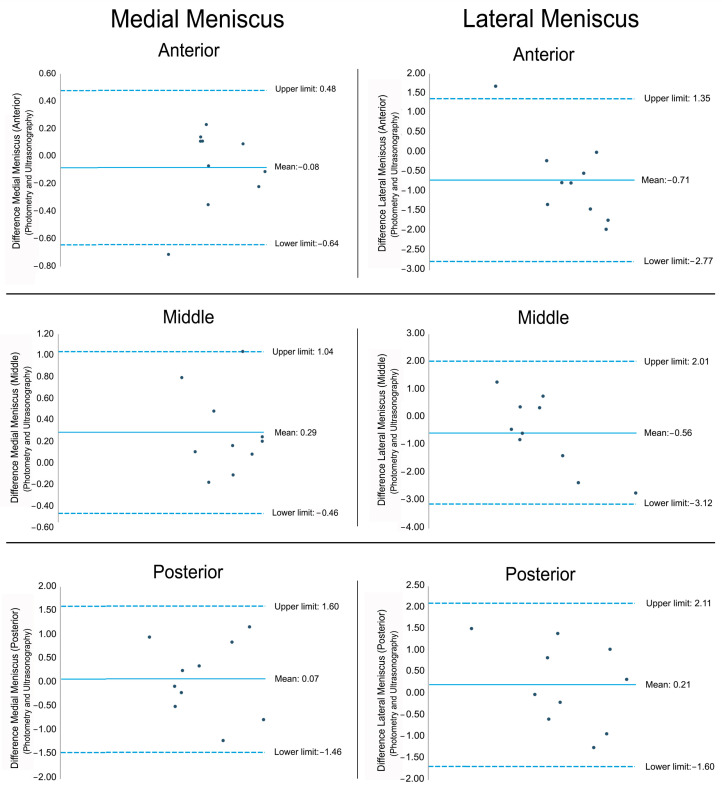
The Bland–Altman plots representing the differences between the mean of the photometry and ultrasonography measurements. The solid line represents the mean difference between the two measurements and the dashed lines represent the upper and lower 95% limits of agreement.

The Bland–Altman plot indicating the differences between photometry and ultrasonography for each part of the medial and lateral meniscus is shown in Figure 6. In the different graphs, we observe that most of the measurements are within the upper and lower limits, which indicates a good agreement.

## 4. Discussion

The findings of our study indicate that ultrasound can visualize a substantial portion of the meniscus, providing results comparable to those obtained through photometry. Both the medial and lateral meniscus were well visualized in all regions, with the ultrasounds showing at least 96.3% meniscal tissue. These results suggest the potential utility of ultrasound in assessing meniscal structures, indicating it may be a reliable tool for capturing the meniscus’s morphology in various anatomical planes. Additionally, this study presents a methodological proposal for meniscal exploration, detailing optimal probe placement in both the longitudinal and transverse planes, as well as the ideal knee positioning to enhance image quality.

The use of ultrasound for evaluating meniscal injuries has a long history, dating back to 1986, when early studies began comparing ultrasound findings with arthroscopic results. However, these initial efforts were limited, as definitive tear characteristics were not clearly identified at that time [14]. Over the past 38 years, significant advancements in ultrasound technology have expanded its application beyond the evaluation of isolated tears. Ultrasound is now used to assess meniscal function and other less common meniscal disorders, such as discoid meniscus, meniscal transplants, and meniscal extrusion [10]. By 2016, ultrasound had also started being used to guide treatments for meniscal pathologies, marking a significant development in its clinical utility [11].

Current studies have shown that ultrasound is capable of visualizing healthy meniscus as triangular, homogeneously hyperechoic structures between the bony cortices of the femur and tibia. The anterior horn, outer rim of the meniscal body, and posterior horn are generally well visualized, although some regions, such as the deep intra-articular portions, are more challenging to assess directly. In previous studies, ultrasound has demonstrated similar sensitivity and specificity to magnetic MRI when evaluated against arthroscopic findings [10]. Systematic reviews have shown pooled sensitivities and specificities for ultrasound in diagnosing meniscal tears, ranging from 77.5% to 88.8% and 83.8% to 84.6%, respectively, compared to MRI and arthroscopy [15]. In addition, some studies have reported that ultrasound demonstrates similar or even superior diagnostic capabilities compared to MRI, particularly when assessed against arthroscopy [16,17]. These findings are particularly evident in individuals with chronic knee pain (over 8 weeks) and in younger patients under 30 years old [17,18]. Although these studies are generally of high quality with low risk of bias, moderate to significant heterogeneity exists, likely due to variations in technique and technology across different studies. Limited data also suggest that inter-observer reliability with ultrasound is moderate, highlighting the need for standardized protocols and further validation [10].

Despite these encouraging findings, ultrasound is not commonly used as a primary diagnostic tool for meniscal injuries and MRI remains the gold standard. This preference persists despite the many advantages of ultrasound, including lower costs, shorter examination times, no contraindications, and the ability to perform dynamic and real-time assessments [19]. This is likely due to the traditional belief that ultrasound cannot fully visualize the entire meniscus, which has historically limited its use in comprehensive meniscal evaluation. There is a lack of studies validating its quantitative assessment against anatomical standards, such as direct dissection. To the authors’ knowledge, no previous studies have specifically aimed to demonstrate the ability of ultrasound to visualize the meniscus, validated through cadaveric dissection. Establishing such validation is crucial for confirming the reliability and accuracy of ultrasound in clinical settings, particularly when used for quantitative assessments of meniscal structures.

Our findings indicate that, upon adjusting for measurement errors in both ultrasonography and photometry, the medial meniscus has a visibility range of 96.3% to 99.2%, while the lateral meniscus achieves visibility exceeding 97.8% in the posterior region. Notably, in the anterior and middle regions of the lateral meniscus, visibility values even surpass 100%, reaching 107.1% and 105.1%, respectively. Despite minor differences, the maximum discrepancy between ultrasound and photometry was only 0.7 mm, indicating a strong agreement between both measurement methods. Several factors may account for the observation that some ultrasonographic measurements exceed 100% of the photometric values. First, the standard deviation of the lateral meniscus measurements obtained via ultrasonography is nearly double that of photometry for the same region, indicating a higher degree of variability inherent to ultrasound measurements. This variability could be attributed to the difficulty in consistently achieving a perfectly perpendicular plane during ultrasonographic imaging, which may result in an overestimation of the meniscus thickness, especially in areas where the meniscus has a complex three-dimensional curvature.

Second, both ultrasound and photometry encounter challenges in isolating the pure fibrocartilaginous tissue of the meniscus without incorporating the adjacent peripheral connective tissues. Specifically, the meniscocapsular attachments, which include the meniscotibial ligaments and the posterior meniscocapsular attachments, could contribute to the overestimation of the meniscus thickness. The meniscotibial ligaments and the posterior meniscocapsular attachments, as detailed in recent anatomical studies, play a critical role in anchoring the meniscus to the tibial plateau and maintaining its position under load [20]. These structures, essential for the stability and function of the meniscus, are closely integrated with the meniscal tissue and may be difficult to exclude from measurements, especially in ultrasonography, where tissue boundaries can be less distinct. Their inclusion in the measurement, whether partial or complete, could lead to an apparent increase in meniscal thickness that exceeds the actual photometric values derived from a direct visual assessment post-dissection.

Thirdly, photometric measurements are obtained from a strictly orthogonal plane (superior view), ensuring direct and consistent measurement of the meniscus without the influence of angulation. In contrast, ultrasonography, despite efforts to achieve a purely orthogonal approach, may involve slight angulation during the procedure. This angulation, combined with the inherent challenges of imaging a curved structure like the meniscus, can extend the measured distance, leading to values that exceed those obtained through photometry. The curvature of the meniscus, particularly in the anterior and posterior horns, further complicates this issue, as it may cause the ultrasound beam to traverse a longer path, effectively increasing the measured thickness. In summary, these factors collectively contribute to the observed discrepancies between photometry and ultrasound, underscoring the inherent challenges and limitations associated with each method in the precise assessment of meniscus visibility. The integration of surrounding connective tissues, the complex curvature of the meniscus, and the technical nuances of ultrasonographic imaging are all critical considerations when interpreting these measurements.

Our study presents a methodological proposal for ultrasound-based exploration of the meniscus, and our results are promising in terms of validating the complete visualization of the meniscus using this technique. Future research should aim to confirm these findings in larger sample sizes to ensure the generalizability of the results. Additionally, it should focus on advanced image processing and analysis technologies to provide a more rigorous assessment, which could enhance the precision of ultrasound measurements in detecting pathological changes in the meniscus. Further studies should explore the inter-evaluator reliability of ultrasound measurements in meniscal assessment. Moreover, refining ultrasound imaging techniques to more effectively differentiate between meniscal tissue and minimize the influence of peripheral tissues is essential.

However, the study is not without limitations. One significant limitation is the small sample size, which may affect the generalizability of the findings. Additionally, the variability in ultrasound measurements, particularly in the lateral meniscus, highlights challenges in achieving consistent results. This variability is further compounded by the challenge of maintaining a perfectly perpendicular plane during imaging, which can lead to overestimation of the meniscal thickness, especially in areas with complex three-dimensional curvatures. Both ultrasonography and photometry face difficulties in isolating the pure fibrocartilaginous tissue of the meniscus without including adjacent connective tissues, such as meniscocapsular attachments. Additionally, the inherent methodological differences between photometry and ultrasound likely contribute to the observed discrepancies, as the orthogonal approach of photometry contrasts with the slight angulation that may occur during ultrasound imaging. This angulation, particularly when imaging the curved meniscus, can result in measurements that exceed those obtained through photometry. Furthermore, this study did not aim to evaluate meniscal pathology using ultrasound but rather to validate its ability to quantify meniscal tissue. While ultrasound has been explored as a tool for detecting meniscal injuries, our findings are limited to its capacity for morphological assessment, and future studies should investigate its potential for diagnosing pathological alterations. Despite these limitations, the study underscores the significant potential of ultrasound as a diagnostic tool for meniscal assessment. Its advantages, including lower costs, real-time imaging, and dynamic assessment capabilities, position ultrasound as a promising technique for the future. With further refinement of imaging techniques and validation against anatomical standards in larger studies, ultrasound could become an even more valuable tool in the precise evaluation of meniscal structures.

## 5. Conclusions

Ultrasound is a valid tool for visualizing the different regions of the meniscus almost in their entirety. The difference between the measurements obtained via dissection and those obtained via ultrasound is less than 0.7 mm. These findings validate the use of ultrasound for the evaluation of the meniscus in both the longitudinal and transverse planes. However, in some cases, a small amount of adjacent connective tissue may be mistakenly identified as meniscal tissue through an ultrasound.

## Figures and Tables

**Table 1:** The test–retest reliability of photometry and ultrasonographic measurements in millimeters.

	Measure 1 Mean ± SD (mm)	Measure 2 Mean ± SD (mm)	ICC (95% CI)	SEM	MDC_95%_
Photometry					
Medial meniscus					
Anterior	8.60 ± 1.00	8.68 ± 0.99	0.992 (0.949; 0.998)	0.009	0.025
Middle	9.40 ± 0.92	9.37 ± 0.85	0.988 (0.954; 0.997)	0.016	0.043
Posterior	10.60 ± 1.31	10.62 ± 1.51	0.967 (0.873; 0.992)	0.069	0.192
Lateral meniscus					
Anterior	10.58 ± 1.73	10.52 ± 1.72	0.997 (0.988; 0.999)	0.007	0.019
Middle	11.18 ± 2.23	11.10 ± 2.19	0.998 (0.989; 0.999)	0.006	0.017
Posterior	10.44 ± 2.00	10.40 ± 1.94	0.998 (0.994; 1.000)	0.005	0.013
Longitudinal Ultrasonography					
Medial meniscus					
Anterior	8.09 ± 0.74	8.10 ± 0.67	0.985 (0.941; 0.996)	0.016	0.044
Middle	8.47 ± 1.08	8.41 ± 0.86	0.938 (0.775; 0.984)	0.089	0.246
Posterior	9.62 ± 1.37	9.47 ± 1.18	0.963 (0.863; 0.990)	0.065	0.180
Lateral meniscus					
Anterior	9.24 ± 1.51	9.25 ± 1.68	0.988 (0.951; 0.997)	0.029	0.080
Middle	9.89 ± 2.21	9.73 ± 1.99	0.983 (0.936; 0.996)	0.049	0.136
Posterior	9.31 ± 1.54	9.31 ± 1.54	0.986 (0.945; 0.997)	0.032	0.089
Transverse section Ultrasonography					
Medial meniscus					
Anterior	8.88 ± 1.09	9.01 ± 1.05	0.929 (0.756; 0.982)	0.107	0.298
Middle	9.71 ± 1.03	9.74 ± 1.03	0.964 (0.863; 0.991)	0.055	0.153
Posterior	11.55 ± 1.95	11.50 ± 1.98	0.990 (0.962; 0.997)	0.029	0.080
Lateral meniscus					
Anterior	11.47 ± 2.40	11.37 ± 2.34	0.992 (0.971; 0.998)	0.026	0.073
Middle	11.77 ± 2.57	11.90 ± 2.60	0.993 (0.972; 0.998)	0.025	0.070
Posterior	11.42 ± 2.75	11.20 ± 2.70	0.995 (0.888; 0.999)	0.039	0.109

Abbreviations: ICC, intraclass correlation coefficient; CI, confidence interval; SD, standard deviation; SEM, standard error of measurement, MDC; minimal detectable change.

**Table 2:** Mean values and difference between photometry and ultrasonography in millimeters.

	Photometry	Ultrasonography	
	Mean ± SD (mm)	Mean ± SD (mm)	Difference
Medial meniscus			
Anterior	8.64 ± 0.99	8.71 ± 0.92	−0.08 [−0.282; 0.129]
Middle	9.39 ± 0.88	9.09 ± 0.93	0.29 [0.014; 0.564]
Posterior	10.61 ± 1.40	10.54 ± 1.49	0.07 [−0.492; 0.630]
Lateral meniscus			
Anterior	9.63 ± 0.86	10.33 ± 1.63	−0.70 [−1.459; 0.039]
Middle	10.27 ± 1.21	10.83 ± 2.24	−0.55 [−1.488; 0.382]
Posterior	10.53 ± 1.54	10.32 ± 1.93	0.21 [−0.484; 0.904]

Abbreviations: SD; standard deviation. The values in brackets represent the 95% confidence interval (CI) of the difference between the photometry and ultrasonography measurements.

**Table 3:** The test–retest reliability of photometry and ultrasonography measurements.

	ICC (95% CI)	SEM	MDC_95%_
Medial meniscus			
Anterior	0.958 (0.845; 0.989)	0.059	0.165
Middle	0.874 (0.468; 0.969)	0.136	0.378
Posterior	0.864 (0.543; 0.965)	0.289	0.802
Lateral meniscus			
Anterior	0.606 (0.047; 0.883)	0.657	1.821
Middle	0.721 (0.250; 0.921)	0.690	1.914
Posterior	0.852 (0.525; 0.961)	0.374	1.035

Abbreviations: ICC, intraclass correlation coefficient; CI, confidence interval; SEM, standard error of measurement; MDC; minimal detectable change; SD, standard deviation.

## Data Availability

Data are available upon request to the corresponding author.
